# Effect of Perioperative Supplementation with Arginine and Omega-3 on Postoperative Complications in Patients Undergoing Gastrointestinal Cancer Surgery: A Pilot Open-Label Randomized Controlled Trial

**DOI:** 10.3390/nu18040651

**Published:** 2026-02-16

**Authors:** Saida Sakhri, Rym Ben Othman, Chaima Jerbi, Halil İbrahim Ceylan, Lamia Naija, Ines Zemni, Henda Jamoussi, Tarek Ben Dhiab, Nagihan Burçak Ceylan, Valentina Stefanica, Ismail Dergaa

**Affiliations:** 1 Department of Surgical Oncology, Salah Azaiez Institute, Tunis 1007, Tunisia; saida.sakhri@fmt.utm.tn (S.S.); lamia.naija@gmail.com (L.N.); ines.zemni@fmt.utm.tn (I.Z.); tarek.bendhiab@fmt.utm.tn (T.B.D.); 2 National Institute of Nutrition and Food Technology, Tunis 1007, Tunisia; benothmanr@gmail.com; 3 Faculty of Medicine of Tunis, University of Tunis El Manar, Tunis 1007, Tunisia; hendajamoussi@gmail.com; 4 Research Unit on Obesity UR18ES01, Faculty of Medicine, University of Tunis El Manar, Tunis 1007, Tunisia; 5 Higher School of Health Sciences and Techniques of Tunis, University of Tunis El Manar, Tunis 1007, Tunisia; chaimajerbi37@gmail.com; 6 Physical Education and Sports Teaching Department, Faculty of Sports Sciences, Atatürk University, Erzurum 25240, Türkiye; 7 Graduate Education Institute, Bayburt University, Bayburt 69000, Türkiye; burcaksehitoglu@gmail.com; 8 Department of Physical Education and Sport, Faculty of Sciences, Physical Education and Informatics, National University of Science and Technology Politehnica Bucharest, Pitesti University Center, 060042 Pitesti, Romania; 9 High Institute of Sport and Physical Education of Ksar Said, University of Manouba, Mannouba 2010, Tunisia; phd.dergaa@gmail.com; 10 Physical Activity Research Unit, Sport and Health (UR18JS01), National Observatory of Sports, Tunis 1003, Tunisia; 11 High Institute of Sport and Physical Education of El Kef, University of Jendouba, Jendouba 7100, Tunisia

**Keywords:** complications, gastrointestinal cancer, immunonutrition, oncological surgery, postoperative outcomes, surgical complications, surgical site infections, wound infections

## Abstract

Background: Perioperative immunonutrition, including arginine and omega-3 fatty acids, has been proposed to support postoperative recovery by modulating immune function. Aim: To evaluate the effects of perioperative arginine and omega-3 supplementation on postoperative infectious complications, mortality, hospital length of stay, intensive care unit duration, and inflammatory markers in patients undergoing gastrointestinal cancer surgery. Methods: In this Pilot Open-Label randomized trial, 35 adult patients scheduled for elective gastrointestinal cancer surgery at Salah Azaiez Institute were randomly assigned to receive either perioperative immunonutrition (n = 18; three daily capsules of omega-3 and one sachet of Arginine+ for 7 days preoperatively and 7 days postoperatively) or standard care (n = 17). Primary endpoints were postoperative infectious complications and 1-month mortality. Secondary endpoints included hospital length of stay, ICU duration, and postoperative biochemical markers. Results: No statistically significant differences were observed between groups in 1-month mortality (*p* = 0.324), hospital length of stay (median 7 vs. 7 days, *p* = 0.392), or ICU duration (median 5 vs. 6 days, *p* = 0.601). Urinary tract infection (5.9% vs. 11.1%, *p* = 0.939) and wound infection rates (5.9% vs. 11.1%, *p* = 0.581) were comparable. Importantly, postoperative C-reactive protein and other inflammatory markers did not differ significantly between groups (CRP: 165 vs. 175 mg/L; intergroup *p* = 0.798). Conclusions: In this trial, perioperative immunonutrition with arginine and omega-3 fatty acids did not improve postoperative clinical outcomes or inflammatory markers in patients undergoing gastrointestinal cancer surgery. At the administered dose and within a small, heterogeneous cohort, immunonutrition did not provide additional benefit beyond standard care. Larger, adequately powered multicenter trials with optimized dosing are required to clarify its role in gastrointestinal oncology.

## 1. Introduction

Gastrointestinal malignancies represent a significant global health burden, accounting for about 25% of cancer diagnoses and nearly one-third of cancer-related deaths [1]. Incidence varies geographically, with colorectal cancer ranking third in incidence and second in mortality globally, whereas gastric and esophageal cancers are more prevalent in East Asia [2]. Surgical resection remains the cornerstone of curative treatment, yet postoperative morbidity and malnutrition continue to challenge outcomes [3,4]. Malnutrition affects 40–80% of patients at diagnosis and is associated with increased complications, prolonged hospitalization, reduced tolerance to oncological therapies, and impaired quality of life [4,5]. Contributing factors include mechanical obstruction, treatment-related adverse effects, metabolic alterations, and systemic inflammation mediated by cytokines such as interleukin-1, interleukin-6, and tumor necrosis factor-alpha, which suppress appetite and induce early satiety [5,6,7]. Upper gastrointestinal cancers are particularly prone to severe nutritional deficits due to obstructive symptoms [8].

Protein-energy malnutrition compromises immune function and amplifies the catabolic response to surgery, increasing the risk of infections, anastomotic leaks, delayed wound healing, and sepsis [9,10,11,12]. Immunonutrition formulas containing arginine, omega-3 fatty acids, glutamine, and nucleotides have been developed to modulate immune and inflammatory responses perioperatively [13,14,15,16]. However, most randomized trials have been conducted in European and North American populations, with limited data from North African and Middle Eastern regions where genetic, dietary, and healthcare delivery patterns differ substantially [17]. Additional challenges include heterogeneity in formulations, dosing, and timing; limited evidence on mortality outcomes; insufficient characterization across cancer types; variable baseline nutritional status; and scarce cost-effectiveness data in resource-limited settings [18,19,20,21,22,23,24].

Preoperative nutritional assessment using validated tools such as the Malnutrition Universal Screening Tool (MUST), Mini Nutritional Assessment (MNA), Nutritional Risk Index (NRI), and Subjective Global Assessment (SGA) is critical for identifying patients at risk and guiding perioperative supplementation. Perioperative immunonutrition enriched with arginine and omega-3 fatty acids may attenuate postoperative inflammation and improve recovery, but evidence from North African and Middle Eastern cohorts remains scarce.

In this context, we conducted a prospective, randomized pilot trial to evaluate the effects of perioperative immunonutrition on postoperative infectious complications, 1-month mortality, hospital length of stay, intensive care unit duration, and perioperative biochemical and hematological parameters in patients undergoing gastrointestinal cancer surgery in Tunisia. Nutritional status was assessed preoperatively using MUST, MNA, NRI, and SGA, and the intervention group received immunonutrition supplementation.

## 2. Materials and Methods

### 2.1. Ethical Approval

This research protocol was approved by the Ethical Committee of the Salah Azaiez Institute under approval number ISA/2023/06. The study was conducted in full compliance with the principles of the Declaration of Helsinki governing human subjects research. This prospective, open-label, randomized controlled pilot trial was registered in the Pan African Clinical Trials Registry under the identifier PACTR202508509050861. All participants provided written informed consent after receiving a comprehensive explanation of study procedures, potential risks and benefits, and their right to withdraw at any time without consequence.

### 2.2. Sample Size Calculation

No formal sample size calculation based on intergroup differences was performed. Although an initial estimate was derived using a single-proportion precision formula, this approach does not provide adequate power to detect differences between two randomized groups.

Therefore, the present study was designed as a pilot randomized controlled trial, with the primary objective of assessing feasibility and safety, and of generating preliminary effect-size estimates, rather than demonstrating definitive clinical efficacy.

Based on institutional feasibility and recruitment capacity, a total sample size of 35 patients was considered sufficient for exploratory analysis. Previous pilot studies evaluating perioperative immunonutrition in gastrointestinal cancer surgery have reported sample sizes ranging from 30 to 60 participants [25,26,27,28,29].

The authors acknowledge that this sample size is underpowered to detect small to moderate differences in postoperative complications and mortality, and that non-significant findings should not be interpreted as evidence of equivalence between groups.

### 2.3. Population

Eligible participants included adult patients aged 18 to 65 years scheduled for elective gastrointestinal cancer resection procedures. Inclusion criteria specified a histologically confirmed gastrointestinal malignancy involving the esophagus, stomach, colon, or rectum; planned curative-intent surgical resection; absence of distant metastatic disease; and ability to tolerate oral intake preoperatively. Exclusion criteria encompassed prior gastrointestinal surgery or conditions affecting nutrient absorption (e.g., short bowel syndrome, inflammatory bowel disease); hepatic failure (abnormal prothrombin time or Child-Pugh class B/C cirrhosis); renal impairment (creatinine clearance < 60 mL/min by CKD-EPI equation); untreated thyroid disorders; ongoing use of omega-3 supplements or other oral nutritional products within 30 days; cognitive deficits interfering with informed consent or protocol adherence; severe systemic infection requiring IV antibiotics; emergency surgical procedures; anticipated inability to tolerate oral intake postoperatively; and voluntary withdrawal from study supplements. Recruitment took place at the Department of Surgical Oncology, Salah Azaiez Institute, Tunis, Tunisia, between October 2023 and March 2024, via systematic screening of the surgical oncology outpatient clinic schedule.

### 2.4. Experimental Design

This study employed a prospective parallel-group design with 1:1 allocation. After providing informed consent and confirming eligibility, participants underwent a baseline assessment that included anthropometry, nutritional screening, quality-of-life evaluation, and biochemical and hematological testing. Patients were randomly assigned, using a computer-generated sequence prepared by an independent statistician, to either perioperative immunonutrition (experimental group) or standard nutritional care (control group). Blinding of patients and clinical staff was not feasible due to the nature of the intervention; however, outcome assessors and data analysts remained blinded to minimize detection bias.

Participants in the experimental group received three daily capsules of Omévie Omega-3 (36% eicosapentaenoic acid and 24% docosahexaenoic acid) and one sachet of Arginine+ (5 g L-arginine) for seven days preoperatively and seven days postoperatively. The control group received a usual normocaloric diet without immunonutrition supplementation. Both groups received identical standard perioperative care, including surgical technique, anesthesia management, postoperative analgesia, thromboprophylaxis, and early mobilization. Research dietitians monitored adherence through twice-daily visits and documentation of consumption.

Follow-up was conducted at three time points: preoperatively (baseline), during hospitalization, and at 30 days postoperatively. Hospital assessments included daily monitoring of vital signs, postoperative complications, and wound status, as well as documentation of adverse events. Postoperative complications were classified according to Clavien-Dindo grading [30]. Outpatient follow-up at one month included a clinical examination and structured telephone interviews for participants who were unable to attend. Serious adverse events occurring more than 30 days after the intervention were documented if related to the intervention.

### 2.5. Assessments

#### 2.5.1. Participant Characteristics

Baseline demographic and clinical characteristics were collected for all study participants at the time of enrollment. These included age, sex, and relevant clinical variables. Tumor location was determined from medical records based on diagnostic investigations performed prior to surgery, such as imaging studies and endoscopic findings. Participants’ personal medical histories were obtained through patient anamnesis and corroborated by review of their medical records. Comorbid conditions of interest at the time of study inclusion, in accordance with standard clinical practice. Dyslipidemia was defined as a documented clinical diagnosis of dyslipidemia in the medical records and/or current use of lipid-lowering medication, e.g., statins, fibrates.

Hypertension and diabetes mellitus were defined as a documented clinical diagnosis in the medical records and/or the current use of antihypertensive or antidiabetic medication at the time of study inclusion. Asthma was recorded as a personal medical history reported by the patient.

Lifestyle factors were recorded based on medical records and patient self-reports. Non-tobacco use was defined as the absence of current smoking or use of tobacco products at the time of hospital admission; this category included both never smokers and former smokers. Similarly, non-alcohol use was defined as the absence of alcohol consumption during the 12 months preceding study inclusion.

#### 2.5.2. Anthropometric Measurements

Body weight and composition were measured via bioelectrical impedance (Tanita BC-418, Tokyo, Japan). Height was measured with a wall-mounted stadiometer (Seca 217, Hamburg, Germany). BMI was calculated as kg/m^2^. Malnutrition was defined as unintentional weight loss ≥ 5% in 1 month or ≥10% in 6 months; severe malnutrition was ≥10% in 1 month or ≥15% in 6 months [31]. Mid-upper arm circumference was measured at the midpoint between the acromion and olecranon.

#### 2.5.3. Handgrip Strength Assessment

Handgrip strength was measured using a CAMRY dynamometer according to standardized protocols [32]. Three maximal efforts of the dominant hand were recorded, with one-minute rest intervals, and the highest value was used. Sarcopenia was defined as <26 kg for men and <16 kg for women [33].

#### 2.5.4. Nutritional Screening Tools

Nutritional status was assessed using four validated screening and assessment tools.

The Malnutrition Universal Screening Tool (MUST) combines the percentage of unintentional weight loss, body mass index (BMI), and the presence or absence of acute disease. MUST is a validated and widely used screening method for identifying the risk of malnutrition [34]. MUST scores were classified as follows: score 0, low risk of malnutrition; score 1, moderate risk of malnutrition; and score ≥ 2, high risk of malnutrition.

The Mini Nutritional Assessment (MNA) is a rapid and reliable tool for evaluating nutritional status. It consists of 18 items assessing health status, mobility, dietary intake, and anthropometric measurements. The MNA was initially developed and validated in geriatric populations [35] but may also be used in patients with cancer. Nutritional status was categorized as malnutrition for MNA scores < 17, risk of malnutrition for scores between 17 and 23.5, and normal nutritional status for scores > 24.

The Nutritional Risk Index (NRI) [36] takes into account changes in body weight and serum albumin levels. It is based on measurements of serum albumin concentration and the ratio of current to usual body weight and is considered a reliable and validated index. The NRI was calculated using the following formula: NRI = 1.519 × serum albumin (g/L) + 0.417 × (current weight/usual weight) × 100.

NRI values > 97.5 indicated no malnutrition, values between 83.5 and 97.5 indicated moderate risk of malnutrition, and values < 83.5 indicated severe risk of malnutrition.

The Subjective Global Assessment (SGA) is a simple, widely used, and validated method for the subjective evaluation of nutritional status. It is based on a structured questionnaire that includes medical history and physical examination. Key parameters include percentage of weight loss, presence of edema, and clinical assessment of muscle mass. The SGA is favored by clinicians for its simplicity, feasibility, and high sensitivity, which is comparable to that of objective nutritional assessment tools [37].

#### 2.5.5. Quality of Life Assessment

The SF-12 questionnaire is a health-related quality-of-life instrument comprising 12 items that measure 8 health domains and assess both physical and mental health [38,39]. The SF-12 evaluates overall health and well-being, including the impact of any illness across a broad range of functional domains. It comprises 12 questions derived from the SF-36 Health Survey and covers the same eight health outcome domains: physical functioning, role physical, bodily pain, general health, vitality, social functioning, role emotional, and mental health.

#### 2.5.6. Biochemical Parameters

Venous blood samples were collected preoperatively and on postoperative day 3 under standardized conditions. Complete blood count (CBC) parameters, including hemoglobin concentration, total and differential leukocyte counts, platelet count, and hematocrit, were analyzed using Sysmex XN-1000, Kobe, Japan; Beckman Coulter DxH 800, Brea, CA, USA; and Mindray BC-6800, Guangdong, China, an automated hematology analyzer based on impedance and flow cytometry principles. Biochemical analyses, including liver function tests (alanine aminotransferase, aspartate aminotransferase, alkaline phosphatase, total bilirubin), serum electrolytes (sodium, potassium, chloride), and C-reactive protein (CRP), were measured using Roche Cobas c501/c701, Basel, Switzerland; Abbott Architect, Abbott Park, IL, USA and Beckman Coulter AU, Brea, CA, USA which employs enzymatic and immunoturbidimetric methods.

#### 2.5.7. Postoperative Complication Assessment

Postoperative complications were systematically documented and classified according to the Clavien–Dindo grading system [30]. Assessments were performed daily during hospitalization and at 1 month postoperatively, either in person or via structured telephone interviews for participants unable to attend in person. Infectious complications, including surgical site infections, pneumonia, urinary tract infections, and intra-abdominal abscesses, were diagnosed using standard clinical, laboratory, and imaging criteria [30].

### 2.6. Statistical Analysis

Data were analyzed using SPSS version 27.0 (IBM Corp., Armonk, NY, USA). Normality of continuous variables was assessed using the Shapiro–Wilk test. Continuous variables were expressed as mean ± standard deviation when normally distributed and as median [interquartile range] when non-normally distributed.

Continuous variables included age, weight, height, body mass index (BMI), C-reactive protein (CRP), alanine aminotransferase (ALAT), aspartate aminotransferase (ASAT), total bilirubin, serum creatinine, length of hospital stay, length of intensive care unit (ICU) stay, and hematological parameters (white blood cells, neutrophils, lymphocytes, and platelets). Categorical variables included sex, comorbidities (hypertension, diabetes, asthma, hyperthyroidism, dyslipidemia), smoking status, alcohol consumption, tumor location, and preoperative treatment.

The following continuous variables were not normally distributed: age, CRP, total bilirubin, ALAT, ASAT, serum creatinine, length of hospital stay, length of ICU stay, and hematological cell counts (PNN, White blood cells, lymphocytes, and platelets).

Between-group comparisons were performed using Student’s *t*-test for normally distributed continuous variables and the Mann–Whitney U test for non-normally distributed continuous variables, as appropriate. Paired comparisons within groups were performed using the paired *t*-test or the Wilcoxon signed-rank test, depending on the distribution of the data; however, interpretation and conclusions were based solely on between-group analyses to avoid within-group significance bias (the difference-in-significance fallacy).

Categorical variables were expressed as counts and percentages and compared using the chi-square test or Fisher’s exact test, depending on expected cell frequencies.

Given the pilot and exploratory nature of the study, no formal adjustment for multiple comparisons was performed, and the study was not powered to test superiority or equivalence between groups; therefore, results should be interpreted with caution. A post hoc power analysis was conducted for the primary outcome to estimate the risk of type II error based on observed effect sizes and the final sample size. A two-sided *p*-value < 0.05 was considered statistically significant.

## 3. Results

### 3.1. Baseline Characteristics of Participants

Thirty-five patients were enrolled and completed the study protocol. Eighteen patients were allocated to the experimental group and seventeen to the control group. Table 1 presents the baseline demographic and clinical characteristics of participants.

Baseline demographic and clinical variables were compared between groups to confirm comparability. No statistically significant differences were observed between the experimental and control groups in terms of age (mean 54.2 ± 8.7 years vs. 52.8 ± 9.3 years, *p* = 0.652) or sex distribution (61.1% male vs. 64.7% male, *p* = 0.812). Comorbidity prevalence, including hypertension, diabetes, and cardiovascular disease, was similar between groups. Lifestyle factors, such as smoking and alcohol consumption, did not differ significantly between groups.

These findings confirm that randomization successfully produced two comparable cohorts prior to intervention.

### 3.2. Cancer Characteristics

Tumor characteristics were well balanced between groups (Table 2). The rectum was the most frequent tumor site, followed by the colon, stomach, and esophagus. All patients underwent complete surgical resection with curative intent. The administration of neoadjuvant chemotherapy, radiotherapy, or combined chemoradiotherapy did not differ significantly between the experimental and control groups, indicating comparable preoperative treatment exposure.

### 3.3. Anthropometric and Nutritional Characteristics

Baseline anthropometric measures, including body weight, height, and body mass index (BMI), were comparable between the experimental and control groups (Table 3), with no statistically significant differences observed. Weight loss was assessed using clinically relevant thresholds commonly used to indicate malnutrition severity. Weight loss ≥ 5% over one month was considered indicative of moderate malnutrition, whereas weight loss ≥ 10% over one month or ≥10% over six months was considered indicative of severe malnutrition. Over the month preceding surgery, weight loss ≥ 5% (moderate malnutrition) was observed in 1 patient (2.85%) in the experimental group and in 2 patients (5.71%) in the control group, while weight loss ≥ 10% (severe malnutrition) was observed in 1 patient (2.85%) in the control group and in none of the experimental group. Over the six months preceding surgery, weight loss ≥ 10% (severe malnutrition) was observed in 4 patients (11.42%) in the experimental group and in 1 patient (2.85%) in the control group, whereas weight loss ≥ 15% (severe malnutrition) was observed in 4 patients (11.42%) in the experimental group and in 5 patients (14.28%) in the control group. Participants who did not meet these malnutrition thresholds are not displayed in the table.

### 3.4. Nutritional Screening Results

Nutritional status was evaluated using multiple validated instruments (Table 4). The Subjective Global Assessment (SGA), Mini Nutritional Assessment (MNA), and Nutritional Risk Index (NRI) showed no statistically significant differences between groups. The Malnutrition Universal Screening Tool (MUST) revealed a statistically significant difference in baseline preoperative nutritional risk, with a higher proportion of patients classified as high risk in the control group than in the experimental group (33.3% vs. 64.7%, *p* = 0.017). Importantly, this difference was present at baseline, before initiation of immunonutrition supplementation, and therefore should not be interpreted as an effect of the intervention. This baseline difference was taken into account when interpreting postoperative outcomes.

### 3.5. Quality of Life Assessment

Baseline health-related quality-of-life scores, assessed using the SF-12, were comparable between groups. Physical Component Summary and Mental Component Summary scores, as well as individual domain scores, showed no statistically significant differences, confirming a similar baseline quality of life between the experimental and control groups. (Table 5).

### 3.6. Biochemical Parameter Changes

Table 6 summarizes postoperative biochemical parameters measured on Day 3. Between-group comparisons revealed no statistically significant differences in serum sodium, potassium, albumin, total bilirubin, ALAT, ASAT, creatinine, or CRP.

### 3.7. Hematological Parameters

Hematological outcomes (Table 7) were analyzed. While postoperative changes in neutrophils, total leukocytes, and hemoglobin were observed within each group, no statistically significant differences were found between the experimental and control groups.

### 3.8. Postoperative Complications

Postoperative complications were classified according to the Clavien-Dindo system (Table 8). No statistically significant differences were observed between the experimental and control groups in overall complication rates, infection rates, or individual complication grades at the one-month follow-up.

### 3.9. Hospital Length of Stay

**Figure 1:**
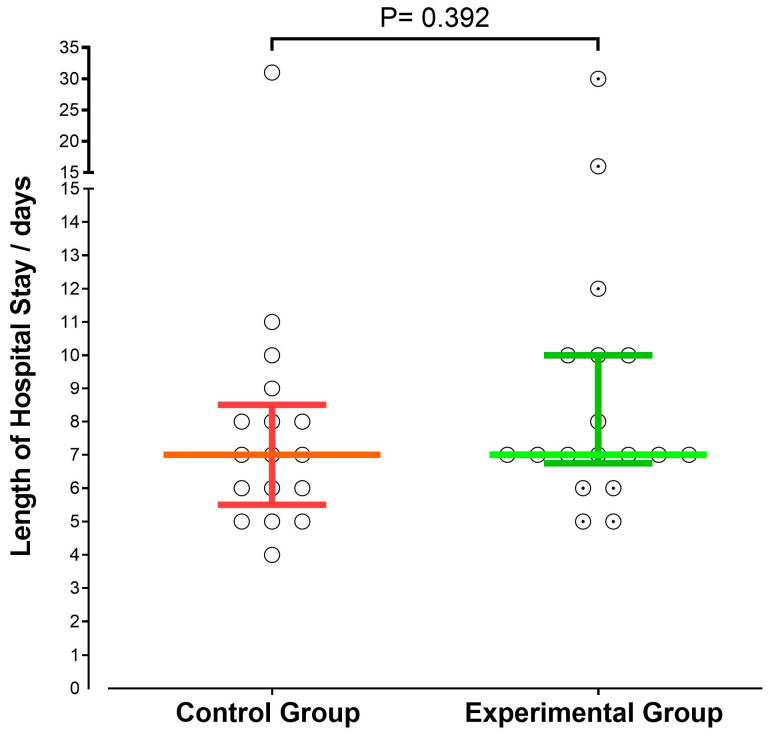
Comparison of hospital length of stay between the two groups. Data are presented as median and interquartile range (IQR). Group comparisons were performed using the Mann–Whitney U test.

No statistically significant difference was observed between the experimental and control groups in hospital length of stay (7 days [IQR: 5.5–8.5] vs. 7 days [IQR: 6.75–10], *p* = 0.392) (Figure 1).

### 3.10. ICU Length of Stay

**Figure 2:**
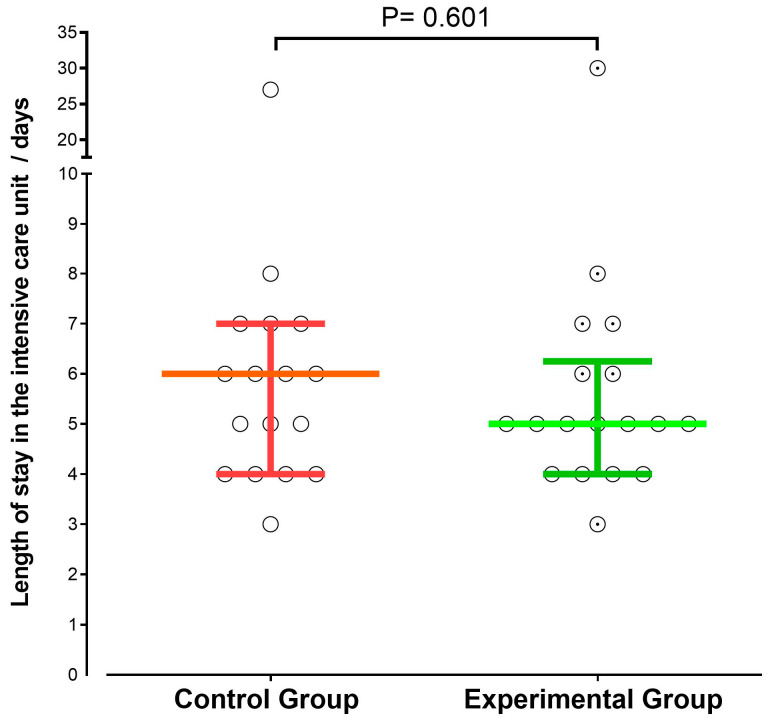
Length of stay in the intensive care unit (ICU) for both groups. Data are presented as median and interquartile range (IQR). Group comparisons were performed using the Mann–Whitney U test.

At one-month follow-up, mortality was rare, with only a single death reported in the experimental group. No statistically significant difference in mortality rates was observed (6 days [IQR: 4 to 7] vs. 5 days [IQR: 4 to 6.25], *p* = 0.601). These findings should be interpreted with caution due to the small sample size of this pilot study (Figure 2).

## 4. Discussion

This controlled, open-label randomized trial evaluated the effects of perioperative immunonutrition on clinical and biochemical outcomes in patients undergoing gastrointestinal cancer surgery. Overall, no statistically significant differences were observed between the immunonutrition and standard care groups regarding postoperative infectious complications, hospital length of stay, intensive care duration, or short-term mortality.

### 4.1. Postoperative Inflammatory Response

Postoperative inflammatory markers, including C-reactive protein, did not differ significantly between the immunonutrition and control groups (intergroup *p* = 0.798), indicating no effect of the supplement on inflammation. Prior studies have suggested that omega-3 fatty acids can modulate inflammatory responses by inhibiting arachidonic acid metabolism and reducing pro-inflammatory eicosanoids, including prostaglandin E2 and leukotriene B4 [17,40,41]. Clinical trials have reported reductions in perioperative inflammatory cytokines with immunonutrition [42,43]. Arginine supplementation may modulate the immune system by enhancing nitric oxide production, which regulates vascular tone, platelet aggregation, and immune cell function [44].

### 4.2. Infectious Complications

Infectious complications occurred in 22.2% of the immunonutrition group versus 29.4% of controls (*p* = 0.607), indicating no statistically significant effect of perioperative immunonutrition on infection rates. Large-scale trials and meta-analyses have previously shown reductions in postoperative infections with immunonutrition [45,46,47]. The lack of significant differences in our study is likely attributable to the small sample size and cohort heterogeneity, and low baseline infection rates, highlighting the limitations of detecting treatment effects in underpowered pilot studies. These observations emphasize that, under contemporary surgical protocols with optimized perioperative care, the incremental benefit of immunonutrition on infection rates may be limited.

### 4.3. Hospital Length of Stay and Intensive Care Duration

Median hospital stay (7 days) and ICU duration (5 vs. 6 days) were not statistically different between groups. Mixed findings in meta-analyses suggest that modest reductions in length of stay may not be detectable in settings where enhanced recovery protocols, early mobilization, structured feeding, and standardized discharge criteria are implemented [43,48,49]. More sensitive metrics, such as time to functional recovery or quality-of-recovery assessments, may better capture subtle benefits, but our study was underpowered to detect such differences.

### 4.4. Baseline Nutritional Status Assessment

The Malnutrition Universal Screening Tool identified higher nutritional risk in controls (64.7% vs. 33.3%, *p* = 0.017), while other assessment tools (SGA, MNA, NRI) showed no significant differences between groups. This finding reflects baseline differences present prior to the intervention and should be taken into account when interpreting postoperative outcomes. The variability across nutritional instruments [50,51,52] highlights the importance of stratified randomization in future trials to balance prognostic factors [53].

### 4.5. Population Heterogeneity and Cancer Type Considerations

The study population included rectum (40%), colon (31%), stomach (23%), and esophageal (6%) cancers, encompassing diverse anatomical sites with different impacts on nutritional status and surgical complexity [8]. Upper gastrointestinal malignancies often present with more severe malnutrition due to mechanical obstruction and dysphagia, whereas colorectal cancers tend to exhibit milder nutritional impairment [44]. Approximately 45% of patients received neoadjuvant therapy, which can further affect nutritional status. This heterogeneity may have diluted potential treatment effects, underscoring the need for future studies to include more homogeneous cohorts or to conduct stratified analyses to better evaluate the impact of perioperative interventions.

### 4.6. Immunonutrition Timing and Duration

Patients received immunonutrition for 7 days preoperatively and 7 days postoperatively, consistent with previous studies [21,54,55,56,57,58]. The study did not demonstrate statistically significant effects of this supplementation on postoperative inflammatory or clinical outcomes. The optimal timing and duration of immunonutrition remain uncertain, and severely malnourished patients may require longer administration or alternative routes to achieve meaningful immunomodulatory effects. Postoperative tolerance to oral supplements may limit adherence, underscoring the need for individualized approaches tailored to nutritional status and gastrointestinal function.

### 4.7. Mortality Outcomes

No statistically significant differences in mortality were observed between groups. At one month, no deaths occurred, and only 1 death was recorded at 3 months in the immunonutrition group (*p* = 0.324). This reflects contemporary surgical techniques, optimized perioperative care, and careful patient selection for curative surgery. Although meta-analyses suggest that perioperative immunonutrition may reduce postoperative morbidity, our study provides no evidence that it affects mortality [45,46,59,60]. Early postoperative mortality is primarily driven by major surgical or oncologic factors rather than minor infections [61,62,63]. Potential immunomodulatory effects on tumor biology would require longer follow-up to evaluate outcomes such as recurrence and overall survival [64].

### 4.8. Study Limitations

This study has several limitations. The single-center design and heterogeneity of gastrointestinal cancer types (colon, rectum, stomach, esophagus) limit generalizability to broader populations and different clinical settings.

Population heterogeneity, including anatomical location and prior treatments, may have diluted intervention effects and precluded meaningful subgroup analyses. Hematological and standard biochemical parameters were assessed, but no immune-specific biomarkers were measured, which limits mechanistic insights. The L-arginine dose (5 g/day) was below the therapeutic range used in pivotal trials (12–18 g/day), which may have limited clinical efficacy.

No adjustment for multiple comparisons was made, and the short one-month follow-up may have missed delayed complications or long-term outcomes, including oncologic recurrence and survival. The lack of cost-effectiveness evaluation also limits conclusions regarding sustainability in resource-limited settings.

Overall, the absence of statistically significant between-group differences indicates that, at the administered dose and within the constraints of this pilot study, perioperative immunonutrition did not demonstrate measurable clinical or biochemical benefits. This study should therefore be regarded as a pilot, hypothesis-generating investigation. Larger, multicenter trials with more homogeneous populations, optimized dosing, and extended follow-up are needed to confirm these preliminary observations.

## 5. Conclusions

This randomized controlled pilot trial among Tunisian patients undergoing gastrointestinal cancer surgery did not show any statistically significant effect of perioperative immunonutrition on postoperative infectious complications, length of hospital stay, intensive care duration, mortality, or biochemical parameters. The study was limited by a small sample size (n = 35), a short follow-up period (1 month), and a relatively low L-arginine dose (5 g/day) compared with doses used in pivotal trials (12–18 g/day). Heterogeneity in cancer types and anatomical locations further limited the ability to detect subgroup-specific effects. These findings highlight the scientific value of null results in a pilot study, demonstrating that, at the administered dose and under the study conditions, perioperative immunonutrition did not provide measurable clinical or biochemical benefits. Future research should focus on adequately powered, multicenter trials with homogeneous patient populations, optimized dosing strategies, longer follow-up, and detailed immunologic assessments (e.g., lymphocyte subsets, cytokine profiles, natural killer cell activity) to better define the potential clinical and mechanistic effects of immunonutrition in gastrointestinal oncology.

## Figures and Tables

**Table 1:** Baseline characteristics of study participants. Data are presented as numbers (percentages) for categorical variables and medians (ranges) for continuous variables.

Characteristic	Experimental Group (n = 18)	Control Group (n = 17)	*p*-Value
Age, years	64.6 (46–75)	60.9 (32–80)	0.333
Sex (Male)	10 (55.6%)	9 (52.9%)	0.812
Hypertension	4 (22.2%)	7 (41.2%)	0.227
Asthma	0	1 (5.9%)	0.296
Diabetes	4 (22.2%)	6 (35.3%)	0.471
Hyperthyroidism	1 (5.6%)	0	0.324
Dyslipidemia	3 (16.7%)	2 (11.8%)	0.679
Smoker	5 (27.8%)	3 (17.6%)	0.691
Alcohol consumption	0	2 (11.8%)	0.229
Tumor location			0.713
Rectum	7 (38.9%)	7 (41.2%)	
Colon	6 (33.3%)	5 (29.4%)	
Stomach	4 (22.2%)	4 (23.5%)	
Esophagus	1 (5.6%)	1 (5.9%)	
Preoperative treatment			1.000
Surgery only	10 (55.6%)	9 (52.9%)	
Chemotherapy	3 (16.7%)	3 (17.6%)	
Radiotherapy	2 (11.1%)	2 (11.8%)	
Chemoradiotherapy	3 (16.7%)	3 (17.6%)	

Age was not normally distributed and was analyzed using the Mann–Whitney U test. Categorical variables were analyzed using the chi-square test or Fisher’s exact test, as appropriate.

**Table 2:** Cancer characteristics and preoperative treatment. Data are presented as n (%).

Characteristic	Experimental Group (n = 18)	Control Group (n = 17)	*p*-Value
Tumor location:			
Rectum	7 (38.9%)	7 (41.2%)	0.713
Colon	6 (33.3%)	5 (29.4%)	
Stomach	4 (22.2%)	4 (23.5%)	
Esophagus	1 (5.6%)	1 (5.9%)	
Preoperative treatment:			
Surgery only	10 (55.6%)	9 (52.9%)	1.000
Chemotherapy	3 (16.7%)	3 (17.6%)	
Radiotherapy	2 (11.1%)	2 (11.8%)	
Chemoradiotherapy	3 (16.7%)	3 (17.6%)	

Data are presented as a number (percentage). *p*-values were calculated using the chi-square test or Fisher’s exact test, as appropriate.

**Table 3:** Baseline nutritional characteristics of patients in the Experimental and Control groups.

Parameter	Experimental Group (n = 18)	Control Group (n = 17)	*p*-Value
Weight (kg)	68.2 ± 13.1	74.5 ± 17.4	0.235
Height (m)	1.66 ± 0.03	1.67 ± 0.01	0.305
Body Mass Index (BMI, kg/m^2^)	24.7 ± 4.3	26.7 ± 6.3	0.289
Weight loss over the last 1 month (%)			
≥5%	1 (2.85)	2 (5.71)	0.603
≥10%	0 (0)	1 (2.85)	0.486
Weight loss over the last 6 months (%)			
≥10%	4 (11.42)	1 (2.85)	0.338
≥15%	4 (11.42)	5 (14.28)	0.711

Data are presented as mean ± standard deviation for continuous variables and as number (percentage) for categorical variables. Continuous variables were compared using Student’s *t*-test. Categorical variables were compared using the chi-square test or Fisher’s exact test, as appropriate. Weight-loss variables represent clinically relevant thresholds used to define malnutrition severity and are not mutually exclusive categories. Weight loss ≥ 5% over one month indicates moderate malnutrition, whereas weight loss ≥ 10% over one month or ≥10% over six months indicates severe malnutrition. Participants who did not reach these thresholds are not displayed.

**Table 4:** Nutritional score results for both groups before surgery.

Score/Category	Experimental Group (n = 18)	Control Group (n = 17)	*p*-Value
Subjective Global Assessment (SGA)			0.295
Without malnutrition	8 (44.4%)	4 (23.5%)	
Moderate malnutrition	8 (44.4%)	12 (70.6%)	
Severe malnutrition	2 (11.1%)	1 (5.9%)	
Malnutrition Universal Screening Tool (MUST)			0.017
Low risk	10 (55.6%)	3 (17.6%)	
Moderate risk	0 (0%)	3 (17.6%)	
Medium risk	2 (11.1%)	0 (0%)	
High risk	6 (33.3%)	11 (64.7%)	
Mini Nutritional Assessment (MNA)			0.097
Normal nutritional status	7 (38.9%)	3 (17.6%)	
At risk of malnutrition	2 (11.1%)	0 (0%)	
Poor nutritional status	9 (50.0%)	14 (82.4%)	
Nutritional Risk Index (NRI)			0.407
Without risk	9 (60.0%)	6 (46.2%)	
Medium risk	5 (33.3%)	7 (53.8%)	
High risk	1 (6.7%)	0 (0%)	

Data are expressed as a number (percentage). All assessments were performed preoperatively, before initiation of immunonutrition supplementation. SGA (Subjective Global Assessment) evaluates global nutritional status based on clinical and dietary history. MUST (Malnutrition Universal Screening Tool) assesses preoperative nutritional risk. MNA (Mini Nutritional Assessment) evaluates nutritional status in adults. NRI (Nutritional Risk Index) is calculated from body weight and serum albumin. P-values were calculated using the chi-square test or Fisher’s exact test, as appropriate; Fisher’s exact test was used when expected cell counts were small.

**Table 5:** Quality of life in patients before immunonutrition.

SF-12 Category	Experimental Group (n = 18)	Control Group (n = 17)	*p*-Value
SF-12 overall health status			0.562
Good health status	6 (33.3%)	6 (35.3%)	
Fair health status	12 (66.7%)	10 (58.8%)	
Poor health status	0 (0%)	1 (5.9%)	

Data are presented as a number (percentage). Comparisons between groups were performed using the chi-square test or Fisher’s exact test, as appropriate.

**Table 6:** Biochemical parameters before and after surgery in patients from both groups.

	Experimental Group			Group Control			
Parameters	Before	After	*p*-Value	Before	After	*p*-Value	*p*-Intergroup
Albumin, g/L	38.6 ± 4.13	34.5 ± 6.50	0.016	35.2 ± 4.34	30.3 ± 2.06	0.001	0.715
Sodium, mmol/L	140 ± 2.54	140 ± 2.37	0.364	138 ± 3.05	140 ± 3.09	0.170	0.396
Potassium, mmol/L	3.99 ± 0.63	3.74 ± 0.54	0.168	3.95 ± 0.41	3.52 ± 0.48	0.005	0.379
Total Bilirubin, (µmol/L)	7.00 (4.60–9.40)	9.10 (7.03–25.4)	0.530	4.95 (4.45–8.18)	11 (7.00–29.0)	0.003	0.086
ALAT, U/L	13.4 (9.30–16.3)	15.9 (11.4–19.3)	0.222	11.6 (8.0–15.4)	15.0 (11.0–21.0)	0.222	0.998
ASAT, U/L	11.6 (8.0–15.4)	15.0 (11.0–21.0)	0.345	13.4 (9.30–16.3)	15.9 (11.4–19.3)	0.222	0.998
Creatinine, µmol/L	56.5 (44.8–73.5)	51.5 (45.0–79.0)	0.343	71.0 (49.0–107)	62.0 (44.5–80.5)	0.162	0.126
CRP, mg/L	66.6 (18.5–100)	165 (128–187)	0.051	11.3 (4.18–158)	175 (139–188)	0.021	0.798

Continuous variables are presented as mean ± standard deviation or median (interquartile range), depending on data distribution. Serum CRP, total bilirubin, ALAT, ASAT, and creatinine were not normally distributed and were analyzed using non-parametric tests. Pre- and postoperative comparisons within each group were performed using the paired Student’s *t*-test or Wilcoxon signed-rank test, as appropriate. Intergroup comparisons were performed using Student’s *t*-test or Mann–Whitney U test on postoperative values. ALAT: alanine aminotransferase; ASAT: aspartate aminotransferase; CRP: C-reactive protein.

**Table 7:** Hematological parameter levels before and after surgery.

	Experimental Group			Group Control			
	Before	After	*p*-Value	Before	After	*p*-Value	*p*-Intergroup
PNN, cells/µL	2480 (649–5565)	6450 (1366–9430)	0.140	4560 (3763–7743)	9075 (6423–11,918)	0.031	0.454
Platelets, ×10^3^/µL	244 (210–276)	251 (180–297)	0.868	260 (233–407)	294 (204–356)	0.130	0.869
WBC, cells/µL	5280 (4603–7655)	8730 (9060–14,440)	0.568	7365 (5710–8410)	12,570 (10,183–13,670)	<0.001	0.753
Lymphocyte, cells/µL	1045 (237–1445)	1005 (700–1645)	0.543	1205 (808–2120)	1040 (590–1295)	0.609	0.201
HB, g/L	12.0 ± 1.38	11.3 ± 1.74	0.010	11.9 ± 1.48	10.8 ± 1.82	0.037	0.457

Data are expressed as mean ± standard deviation or median (interquartile range), according to data distribution. Hematological cell counts were not normally distributed and were analyzed using non-parametric tests, whereas hemoglobin was normally distributed and analyzed using parametric tests. Pre- and postoperative comparisons within groups were performed using the paired Student’s *t*-test or Wilcoxon signed-rank test, as appropriate. Intergroup comparisons were performed using Student’s *t*-test or Mann–Whitney U test.

**Table 8:** Postoperative complications according to Clavien-Dindo in patients at one month after surgery.

	Experimental Group n = 18	Control Group n = 17	*p*-Value
Score Clavien Dindo			
Grade I	12 (66.7%)	12 (70.6%)	0.555
Grade II	5 (27.8%)	4 (23.5%)	
Grade IV	0 (0%)	1 (5.9%)	
Grade V	1 (5.6%)	0 (0%)	
Infection complications	1 (5.6%)	2 (11.8%)	0.603
Urinary infection	3 (16.7%)	3 (17.6%)	0.939
Surgical site infections	2 (11.1%)	1 (5.9%)	0.581

Data are expressed as a number (percentage). *p*-values were calculated using the chi-square test or Fisher’s exact test, as appropriate; Fisher’s exact test was used when expected cell counts were small.

## Data Availability

The datasets generated and/or analyzed during the current study are available in the Figshare repository under the DOI 10.6084/m9.figshare.30727319. Additional materials can be provided by the corresponding author upon reasonable request.
